# Progress in Ophthalmology

**Published:** 1901-08-17

**Authors:** 


### Progress in Ophthalmology.

**Visual Changes in Acromegaly.**—W. A. Holden,[1] of New York, has collected 200 published cases of acromegaly, and states that in about half of them visual disturbances have been reported. Out of these cases about 50 per cent. have shown concentric contraction of the visual field, with diminution of central acuteness of vision. In somewhat less than 50 per cent. there has been bi-temporal hemianopsia, absolute or for colours only, with or without contraction of the nasal halves of the fields. In half-a-dozen cases there has been homonymous hemianopsia, absolute or for colours only, and in one case there was found to be bi-nasal hemianopsia. It appears there is no special time in the course of the disease for the appearance of these visual symptoms. They come on, occasionally, soon after the enlargement of the extremities is noticed, but usually not until years afterwards. The disease may exist 10 or 15 years without the appearance of any visual disturbance whatever.

**Hæmorrhage from the Conjunctiva of an Infant.**—Yet another case of this malady is reported by Hansell,[2] of Philadelphia, in a child admitted to his hospital on July 9th and who died on August 19th. The primary diagnosis was marasmus, in a seven months child, who was admitted when one day old and weighed 3lb. 3½oz. The father and mother were living and apparently in good health. The mother bled excessively from very small wounds. Besides this there was no definite history of hæmophilia in the parents, brothers, or sisters. When three days old the patient's left eye became swollen, and discharged a small amount of thick pus. The cornea was partly opaque. The purulent discharge ceased in a few days, after irrigation and iced compresses, and was replaced by a bloody discharge of serum, and at times of almost pure blood, which seemed to ooze from the conjunctiva. The lids were slightly swollen, and the conjunctiva red and velvety. From July 25th to 30th there was a constant discharge of blood, apparently from the conjunctival surface of the upper lid. The blood seemed to come from the whole surface, and was sufficiently abundant to overflow from the commissure on to the face. The hæmorrhage ceased, and swelling disappeared after a few days' treatment with solution of alum. A small superficial ulcer now appeared on the cornea about the size of a pin's head. The blood count showed 5,540,000 red corpuscles and 5,600 white corpuscles, but enough blood could not be obtained to test the percentage of hæmoglobin. Although the child was kept in an incubator, at a temperature of 99°, and received nourishment and stimulation, it gradually wasted until at its death it only weighed 2¾ lbs. Hansell in discussing the cause of this condition in his own case thinks it was the result of the exceeding debility of the infant, and the preceding mild attack of purulent conjunctivitis. He thinks that hæmophilia might be excluded as a cause.

**Bacteriology of the Conjunctival Sac.**—In an article, the result of an exhaustive examination on this subject, Jamieson[3] says: As far as external conditions are concerned there seemingly could be no better "anatomical sink" for the collection and propagation of bacterial life than the conjunctival sac. This is strong language to use, and shows the necessity for giving the matter more consideration than it generally receives. There are great divergencies of opinion as to the proper treatment of the conjunctival sac prior to operation, and the consequences are so extremely grave, if anything goes wrong, that it is well if one can come to a thorough understanding on the subject. Dr. Jamieson's observations are based on secretions taken from 50 eyes, presenting the average normal degree to be found in large cities, where the dust, debris, and infective material in the atmosphere, must of necessity produce a slight degree of hyperæmia. Only 13 out of the 50 secretions were found to be sterile. The plates containing the infected cultures, had a varying number of colonies, ranging from 1 or 2 minute particles to 10, 20, or 30 colonies. The bacteria found in the sacs, and isolated, were staphylococcus pyogenes aureus, staphylococcus pyogenes albus, staphylococcus epidermis albus, aero bacillus citreous, xerosis bacillus, bacillus coli communis, bacillus subtilis. Two of the yeast variety of saccharomyces and some others were found, but were not identified as they presented no pyogenic properties. The point which he emphasised was the presence of the pus-producing organisms in the practically normal conjunctival sac. The staphylococcus pyogenes aureus was found twice. The staphylococcus pyogenes albus, and its kindred organisation, the epidermis albus of Welsh, were found in 16 out of the 50 cases, the pyogenes albus constituting seven of that number. Of these organisms the former is more virulent than the latter. That pyogenic bacteria are found in the conjunctival sac has been the experience of most observers. As opposed to this apparent fact, Jamieson mentions the following contradictions:—(1) The comparative rarity of suppurative conjunctival conditions, taking the population as a whole. (2) The relative infrequency of suppuration after operative interference even under adverse circumstances. (3) The large proportion of sterile conjunctivæ found in the bacteriological investigations of most observers. He believes there must be some rational explanation of this immunity and notes these explanations under the following head. (1) The property of the normal conjunctival epithelium to resist the inroads of infection. (2) The special property the phagocyte possesses, in antagonising pyogenic or other bacterial life. (3) The properties of the lacrymal and conjunctival secretion itself, when normal, of inhibiting growth of germ life. (4) The irrigative apparatus by means of which the eye is constantly douched by a steady flow from the lacrymal gland to the nasal duct. It has been supposed that on account of the relative sterility of the lacrymal sac in a large proportion of cases, the secretion possessed in itself some antiseptic properties capable of inhibiting germ life, but the writer collected some 30 m.m. of human tear secretion; with this he made a series of experiments, and from these deduces the fact that while tears are not the best medium for germ life, they are capable of sustaining some forms for a certain period, while other forms seemingly flourish continually in it. He summarises his points thus: (1) That pus-producing organisms are found in the normal conjunctival secretion, although probably in an attenuated form; (2) That under normal conditions they do not propagate; (3) That the eye under normal conditions is bountifully supplied with means of antagonising bacterial growth; (4) That diminished resistance, such as occurs in inflammation of the membrane in operative interference, alters the nutritive value of the secretion and probably converts it into a more suitable media for germ life; (5) That the secretion in front of the eye is not an antiseptic in itself; (6) That strong antiseptics in the conjunctival sac diminish the resistance, and place the eye on a lower plane to resist germ invasion; (7) That great attention should be given to washing out the residual bacteria prior to operation; (8) That as much care should be taken in regard to antiseptics and cleanliness in the external preparation of the patient and operator as is adopted by the general surgeon of modern times, inasmuch as while the danger of suppuration is more remote the result, if it occurs, is more dangerous.

**Tincture of Iodine in Dendritic Ulcer of Cornea.**—The treatment of dendritic keratitis is so poor in its results that one rather welcomes any new addition to their store of remedies. Now Friedenwald[4] recommends tincture of iodine. He has used it in 25 cases of dendritic keratitis and marginal ulcer of cornea, and he says in no case has it failed to bring relief and without untoward symptoms. The usual method of applying remedies to these ulcers is used, namely, cotton-wool wrapped tightly round a fine wood point so as to form a narrow firm swab; this is dipped into the tincture, and the excess having been shaken off, the ulcer—previously prepared by instillation of cocain and fluorescin—is thoroughly scrubbed until a distinct brown discolouration is seen. The neighbouring epithelium is much loosened and curls up in every direction. It is important to touch this, and especially the minute infiltrations seen a millimetre or two away from the main ulceration, for the progress of the disease is such that once these infiltrations are seen, the furrowed ulceration soon makes its appearance. He thinks the only error that can be made is to apply the iodine too cautiously, and has never seen any bad results from its being applied too freely. The application is followed by a good deal of pain, lasting some hours. The eye is bandaged and a boric acid ointment used, the bandage is dispensed with in a few days. Some little time ago H. R. Swanzy, of Dublin, advocated scrubbing these ulcers with absolute alcohol, with which he had brilliant results, but when we last saw him about six months ago he said he had given up the alcohol and now used the sulphate of copper crystal, which, being sharpened to a fine point was handier than alcohol and gave just as good results. The main thing in all these various forms of treatment seems to be—to get your remedy well in under the overhanging edge of the furrow, as it is undoubtedly here that the bacteria lurks. On this account the fluorescin is so useful, as it shows up affected places that would otherwise be quite invisible and would be likely to escape treatment.

[1] Amer. Jour. of Med. Sci., April 1901. [2] Quar. Med. Jour., May 1901.

[3] Quar. Med. Jour., May 1901. [4] Amer. Jour. of Med. Sci., April 1901.

